# Detection of the Plant Pathogen *Pseudomonas Syringae* pv. *Lachrymans* on Antibody-Modified Gold Electrodes by Electrochemical Impedance Spectroscopy

**DOI:** 10.3390/s19245411

**Published:** 2019-12-09

**Authors:** Zofia Cebula, Sabina Żołędowska, Karolina Dziąbowska, Marta Skwarecka, Natalia Malinowska, Wioleta Białobrzeska, Elżbieta Czaczyk, Katarzyna Siuzdak, Mirosław Sawczak, Robert Bogdanowicz, Dawid Nidzworski

**Affiliations:** 1 Institute of Biotechnology and Molecular Medicine, 3 Trzy Lipy St., 80-172 Gdańsk, Poland; 2 SensDx, 14b Postępu St., 02-676 Warszawa, Poland; 3 Polish Academy of Sciences, The Szewalski Institute of Fluid-Flow Machinery, The Centre for Plasma and Laser Engineering, 14 Fiszera St., 80-231 Gdańsk, Poland; ksiuzdak@imp.gda.pl (K.S.); mireks@imp.gda.pl (M.S.); 4 Faculty of Electronics, Telecommunications and Informatics, Department of Metrology and Optoelectronics, Gdańsk University of Technology, 11/12 G. Narutowicza St., 80-233 Gdańsk, Poland

**Keywords:** immunosensor, gold electrode, electrochemical impedance spectroscopy, cyclic voltammetry, plant pathogen, *Pseudomonas syringae* pv. *lachrymans*

## Abstract

The present work describes an impedimetric immunosensor for *Pseudomonas syringae* pv. *lachrymans* (*Psl*) detection. This pathogen infects many crop species causing considerable yield losses, thus fast and cheap detection method is in high demand. In the assay, the gold disc electrode was modified with 4-aminothiophenol (4-ATP), glutaraldehyde (GA), and anti-Psl antibodies, and free-sites were blocked with bovine serum albumin (BSA). Sensor development was characterized by cyclic voltammetry (CV) and antigen detection by electrochemical impedance spectroscopy (EIS) measurements. Seven analyzed strains of *Psl* were verified as positive by the reference method (PCR) and this immunoassay, proving sensor specificity. Label-free electrochemical detection was in the linear range 1 × 10^3^–1.2 × 10^5^ CFU/mL (colony-forming unit) with an R^2^ coefficient of 0.992 and a detection limit (LOD) of 337 CFU/mL. The sensor did not interfere with negative probes like buffers and other bacteria. The assay was proven to be fast (10 min detection) and easy in preparation. The advantage was the simplicity and availability of the verified analyte (whole bacteria) as the method does not require sample pretreatment (e.g., DNA isolation). EIS biosensing technique was chosen as one of the simplest and most sensitive with the least destructive influence on the probes compared to other electrochemical methods.

## 1. Introduction

*Pseudomonas syringae* species divide into 60 subtypes (pathovars) basing on pathogenic characters, and *lachrymans* is one of them [1,2]. These Gram-negative bacteria are responsible for diseases of many crop species causing spot, speck, and blight diseases [3]. The main hosts are cucumbers, tomato, apples, olives, oats, rice, flowers, and more [4]. Angular leaf spot (ALS) is a widespread cucumber disease caused by this phytopathogen, limiting its open-field production [5,6,7]. The symptoms are water-soaked lesions on the leaves, and when they become necrotic contribute to the reduced photosynthesis capacity [8,9]. The danger in *lachrymans* pathovar is also in possibilities to facilitate other infections, like *Pseudoperonospora cubensis*, which is the most destructive cucumber pathogen [10].

ALS causes severe yield losses, estimated on a 30%–60% level in fruits depending on the species susceptibility [11]. From an economic point of view, uninfected cucumber farming is very crucial, as it reaches 2.1 million hectares with a production of 71.3 million tons in China, USA, and EU [10]. Unfortunately, only partial resistance to *Pseudomonas syringae* pv. *lachrymans* (Psl) was confirmed in the genus Cucumis [12]. There is no method for direct protection against this pathogen. None of the chemical pesticides can cure the crop of the bacterial disease. Chemical treatment is ineffective, and the most common copper treatment decreases crop quality [11,13].

Except for ALS prevention, important is its detection in the early stages of infection. Currently, developed bacterial detection methods focus on the molecular level, mainly sequencing and PCR-based methods. *P. syringae* genome varies significantly between pathovars [14]. Conventional *P. syringae* pathovars detection bases on pathogenicity tests [15,16,17] or LOPAT tests [18] which are a series of tests determining: Levan production (L), oxidase production (O), pectinolytic activity (P), arginine dihydrolase production (A), and tobacco hypersensibility (T). Besides that, molecular methods offer ELISA analysis [19,20,21] and PCR [22,23,24,25]. Direct detection of *lachrymans* pathovar focuses on similar methods. Literature reports show multi-locus sequence typing [26], rep-PCR [15], quantitative real-time PCR [27], and loop-mediated isothermal amplification [28]. Immunological methods like ELISA [29] are also known. The detection systems mentioned above are expensive, labor intensive, and time consuming.

Fast and easy methods for plant pathogen detection are in high demand. Promising are optical and electrochemical assays as they offer automatization and miniaturization possibilities [30].

Biosensing techniques focus mainly on antibody-based and DNA-based platforms [31]. They are willingly applied in point of care (POC) devices for bacteria detection using optical, magnetic, or electrical visualization [32]. They allow reducing analysis costs, shortening the detection time, and excluding the qualified staff. Jarocka et al. detected prunus necrotic ringspot virus (PNRV) in cucumber leaf extracts using the electrochemical method. The authors modified glassy carbon electrode (GCE) with protein A for antibodies immobilization and were able to detect PNRV in 10,000-time dilutions [33]. Malecka et al. proposed the DNA-hybridization method for label-free quantification of plum pox virus (PPV) being the most widespread disease of European stone fruits. GCE was modified with complementary DNA to PPV, and voltammetric measurements allowed to detect PPV at 10–50 pg/mL level with a detection limit (LOD) of 12.8 pg/mL in plant extracts [34]. Most often developed optical immunosensors are paper-based lateral flow devices. Drygin et al. evaluated the test for potato virus x (PVX). Infected leaves and sprouts were tested. The authors used colloidal gold for assay visualization. Fast detection (15 min) in non-clarified leaf extracts showed LOD of 2 ng/mL of PVX [35]. Zhao et al. developed a dipstick DNA sensor for *Acidovorax avenae* subsp. *citrulli* (ACC) detection. ACC infects mainly cucurbit. Similarly to Drygin et al., the gold nanoparticles were used as a label but applied on oligonucleotide samples. The LOD of the assay was 4 nM of bacterial DNA [36].

In the case of Psl, except for gold-standard detection methods, there is no research about magnetic, colorimetric, and electrochemical detection of *Psl*. Innovatory is the application of gold nanoparticles (AuNPs) in the detection system for signal enhancement. Vaseghi et al. (2013) functionalized DNA with AuNPs for colorimetric detection of *P. syringae* 24 isolates [37]. Lau et al. (2017) combined the recombinase polymerase amplification method with AuNPs conjugation for voltammetric detection of *P. syringae* on carbon electrodes [38].

Genosensors, compared to immunosensors, are believed as very stable and easy in synthesis and storage [39]. However, the main disadvantage is that they require a DNA sample which (when it is not synthetic [40]) needs to be prepared in an amplification method, e.g., PCR. In the case of bacteria detection, the sample must be pretreated to release the genomic DNA. Electrochemical immunosensors detect antigen–antibody interactions on the transducer surface, which generate electrochemical signals [41]. The general concept of the method is similar to conventional ELISA, but often offering higher sensitivities thanks to advanced transducer technology [42]. Antibody-based biosensors can detect both matrix or surface proteins of the target pathogen. In the case of external proteins, practically no sample preparation is needed, which is the main advantage over genosensors.

From electrochemical measurement methods, electrochemical impedance spectroscopy (EIS) is favorable thanks to real-time reaction monitoring, various data generation (about the electrode structure, and chemical and psychical changes during analyte binding), being label-free, and having low-current requirements and a nondestructive impact on the analyte [43].

In this work, we want to present an alternative sensor for DNA-based sensors detecting *Psl* robustly and accurately. The label-free electrochemical detection of *Psl* on gold electrodes modified with anti-Psl antibodies was not previously reported. The immunosensor was proven to be fast (10 min detection) and easy in preparation. The advantage is the simplicity and availability of the verified analyte (whole bacteria). The assay does not require DNA isolation or any sample pretreatment. The electrochemical impedance spectroscopy method was chosen as it is not destructive for the analyte.

## 2. Materials and Methods

### 2.1. Materials and Reagents

All chemicals were used without further purification. Aqueous solutions were made using double-distilled sterile water (ddH_2_O). Ethanolic and aqueous solutions were deaerated using argon from Air Products (Poland) and stored in 4 °C conditions. For electrochemical measurements, potassium hexacyanoferrate(II), potassium hexacyanoferrate(III), and potassium chloride were purchased from Chempur (Piekary Śląskie, Poland). The working gold electrodes were purchased from Mineral (Poland), the reference silver chloride electrode was purchased from Mineral (Łomianki-Sadowa, Poland), and a platinum sheet was the counter electrode. For electrodes preparation and modification, 99.8% ethanol and sulfuric acid were provided by Chempur (Piekary Śląskie, Poland); phosphate-buffered saline (PBS) tablets, 97% 4-ATP, 25% GA, and Bovine Serum Albumin (BSA) were provided from Sigma Aldrich (Munich, Germany). The anti-Psl polyclonal antibody was purchased from Creative Diagnostics (USA). For bacteria growth, Yeast extract – Peptone – Glucose – Agar (LPGA) medium, peptone, yeast extract, and agar were provided by Sigma Aldrich (Munich, Germany). Seven strains of *Psl* were purchased from CIRM-CFBP (Beaucouzé, France) and are described in Table 1. Genomic DNA was isolated with the ExtractMe DNA Bacteria kit from Blirt (Gdansk, Poland). The primers were synthesized by Sigma Aldrich (Munich, Germany), and for qPCR amplification, the SensiFAST SYBR No-ROX kit from Bioline (London, UK) was used.

### 2.2. Bacterial Samples Preparation

The bacteria were firstly plated on solid LPGA medium (glucose 10%, peptone 5%, yeast extract 5%, agar 16%, pH 7.5). Afterward, single bacterial colonies were inoculated in LPGA (without agar) in order to obtain overnight cultures. The overnight cultures were centrifuged 6500 RPM, 10 min. Bacterial pellets were resuspended in 1 mL sterile ddH_2_O, and OD_600_ was measured. The bacterial inoculums were adjusted to OD600 approx. 0.1, which roughly equals 0.7 × 10^7^ CFU/mL (colony-forming unit).

### 2.3. Bacterial Samples Verification and Quantification

**Figure 1:**
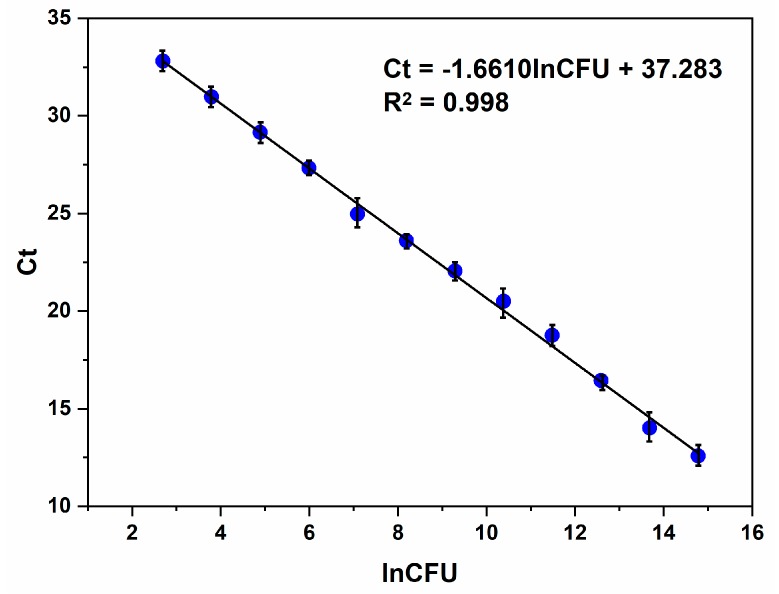
The standard curve of *Pseudomonas syringae* pv. *lachrymans* (*Psl*) bacteria quantification from qPCR.

The *Psl* was identified in samples by qPCR according to Gazdik et al. [27] and based on a sequence of the *nfrB* gene. A 2 µL of sample was added to reaction mixture consisting of 5 µL 2×SensiFast Sybr No-Rox Mix, 1 µL of each primer (10 µM): Pal-nfrB-fwd (5′-GTA CGG TAT GCA AGC GAT CTTC-3′) and Pal-nfrB-rev (5′-CCG AAA CAG GTA ACG GT-3′) [27], and 1 µL of water. Quantification of bacteria was based on a standard curve made of three-fold dilutions of genomic DNA of the reference strain *Psl* 1729 and is presented in Figure 1. The genomic DNA was isolated according to the ExctractMe Bacterial DNA kit protocol.

### 2.4. Apparatus

Cyclic voltammetry and EIS measurements were carried out on PalmSens4 potentiostat (Netherlands) with PSTrace 5.6 software. For bacteria preparation, Thermo Scientific Pico 17 (USA) was used, and OD_600_ was measured on Biosan DEN-1 Biogenet (Poland) densometer. The qPCR reaction was carried out in Bio-Rad CFX Connected thermocycler (USA). The DNA concentration was measured by Thermo Scientific NanoDrop One.

### 2.5. Electrochemical Measurements

All electrochemical measurements were performed using a three-electrode system. Gold discs of 3-mm diameter (ca. 0.0707 cm^2^ surface area) served as working electrodes, silver chloride electrode (Ag/AgCl/3 M KCl) was used as a reference electrode, and a platinum sheet was a counter electrode. Cyclic voltammetry (CV) measurements were carried for gold modification steps evaluation (Figure S1 in the Supplementary Materials), and EIS measurements were carried for both gold modification steps evaluation and bacteria detection. The supporting electrolyte was 5 mM K_3_[Fe(CN)_6_]/K_4_[Fe(CN)_6_] in 0.1 M KCl aqueous solution and used for both CV and EIS experiments. Before use, the electrolyte was deaerated using argon stream. CV data were collected in the voltage window of −0.5 to +0.5 V at the scan rate of 100 mV/s, always in triplicate. The impedance spectra were recorded in frequencies between 10 kHz and 0.5 Hz (21 steps). The applied potential value was formal potential (E_f_) +55 mV (held for 45 s before measurement) and a sinusoidal potential of 10 mV amplitude. All experiments were done in triplicate. Measurement data were analyzed using EIS Spectrum Analyser [44].

### 2.6. Preparation of Electrodes

Before use, the gold electrodes were rinsed with ethanol and dried in the argon stream. Further, they were cleaned with CV (30 scans in 0.5 M H_2_SO_4_ in the potential range from −0.1 to +1.5 V with the scan rate of 100 mV/s) and tested by CV and EIS measurements to ensure the acceptable electrochemical parameters (Z’ of 150 Ω in EIS and redox peaks ΔE of 110 mV in CV).

### 2.7. Fabrication of Electrochemical Immunosensor

After electrodes cleaning, they were rinsed with ethanol and incubated in 0.1 M ethanolic solution of 4-Aminothiophenol (4-ATP) for 17 h in 4 °C conditions. The unbound particles were flushed away with pure ethanol, and the gold surface was dried in an argon stream. In the next modification step, the 2.5% aqueous solution of GA was placed on the electrode and left for 15 min in a dark place (room temperature). The excess of GA was profusely rinsed with ddH_2_O, and the surface was dried using argon. On the gold electrodes modified with 4-ATP-GA linker, the anti-Psl antibodies diluted 1000-times (according to the producer information) in 1 × PBS pH 7.4 were placed for 1.5 h and incubated in 4 °C conditions. After this step, the electrodes were gently rinsed with a small amount of 1 × PBS pH 7.4 and left wet to avoid the structure disruption. The last step of modification included free-sites blockage. The 1% aqueous solution of BSA was placed on antibodies-modified gold and left for 30 min incubation in 4 °C conditions. The sensor prepared according to the above procedure was flushed gently with 1 × PBS pH 7.4 and stored in stable conditions (100% relative humidity, 4 °C) before use.

## 3. Results

### 3.1. The Sensing Principle of Biosensor

**Scheme 1:**
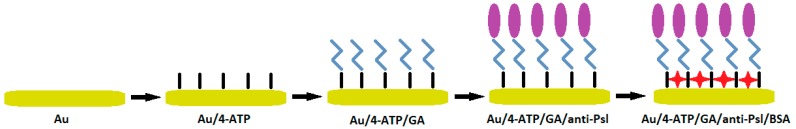
Immunosensor preparation steps. Abbreviations: Au, bare gold electrode; Au/4-aminothiophenol (4-ATP), electrode modified with 4-ATP; Au/4-ATP/ glutaraldehyde (GA), electrode modified with 4-ATP and glutaraldehyde; Au/4-ATP/GA/anti-Psl, electrode modified with 4-ATP, glutaraldehyde, and anti-Psl antibodies; Au/4-ATP/GA/anti-Psl, electrode modified with 4-ATP, glutaraldehyde, and anti-Psl antibodies; Au/4-ATP/GA/anti-Psl/ bovine serum albumin (BSA), electrode modified with 4-ATP, glutaraldehyde, anti-Psl antibodies and BSA.

The immunosensor preparation procedure is presented in Scheme 1. In the first modification step, the thiol groups of 4-ATP were chemisorbed on the gold surface in a structurally well-defined layer. This spontaneous but stable organization of molecules on solid structures is called self-assembled monolayer (SAM). On the top of SAM, the amine groups of 4-ATP remained free. The second modification step involved the amide bond formation between 4-ATP and GA. GA is a cross-linking reagent that binds amine groups. After modification, the formed surface was aldehyde-ended, which can further bind proteins. The next step was anti-Psl antibodies linkage on the top of Au/4-ATP/GA. The last step was for the exclusion of any unspecific reactions between electrode free-sites and antigen. The neutral, non-reactive BSA protein was used as a blocking reagent. All detailed information about the modification condition is described in the Materials and Methods section.

### 3.2. Electrochemical Characterization of Biosensor

**Figure 2:**
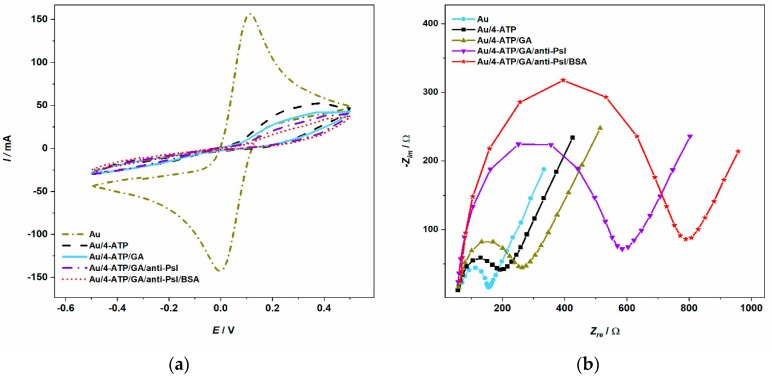
Measurements of biosensor fabrication steps: (**a**) Cyclic voltammetry (CV) spectra for the bare and modified Au electrode, (**b**) electrochemical impedance spectroscopy (EIS) spectra for bare and modified Au electrode. Registered in 5 mM K_3_[Fe(CN)_6_]/K_4_[Fe(CN)_6_]/0.1 M KCl.

The fabrication process of *Psl* biosensor was characterized by CV and EIS measurements. CV records indicated the charge transfer changes, and EIS records showed the resistance changes on the electrode surface. According to Figure 2a, the CV spectra of the bare Au electrode showed its reversible behavior towards active redox species used in the electrolyte ([Fe(CN)_6_]^3-/4-^). The potential difference (∆E) between reduction and oxidation reactions was 110 mV with current heights of 120 mA and 132 mA for cathodic and anodic peak, respectively. The 4-ATP chemisorption resulted in the drastic decrease of this characteristic, proving the electron transfer blockage between the electrolyte and the electrode. No characteristic maximum of the cathodic current peak was notable, and the anodic peak moved towards higher potentials. The following stages of protein linkage to the gold platform resulted in the successive lowering of anodic currents. Simultaneously, the EIS spectra (Figure 2b) for the bare gold electrode showed the standard behavior of the electrochemical system. The Nyquist plot (Z’(Z’’) function) indicated both the interfacial electron-transfer kinetics and redox probe diffusion, which determined the general electrode process. This characteristic is defined as Faradaic impedance, where the curve is semicircle in the higher frequencies’ region (kinetics) and straight in the lower frequencies (diffusion) [45]. The SAM of 4-ATP significantly changed the EIS characteristics, increasing the impedance values but remaining in kinetics-diffusion rate. In the next steps, the linkage of GA, similarly, the increase of impedance was observed. These changes were relatively small compared to the next steps. Proteins immobilization (anti-Psl and BSA) showed a drastic rise in the semicircle diameter.

**Scheme 2:**
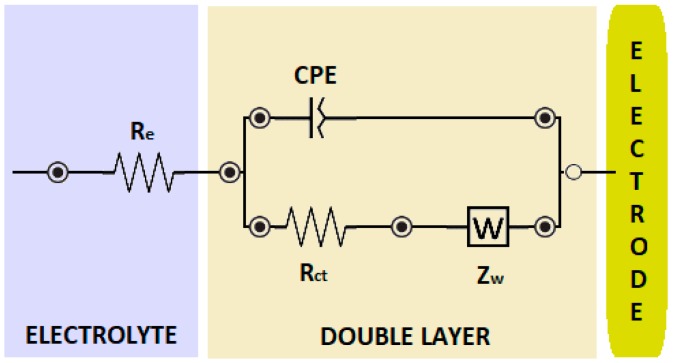
Electric equivalent circuit model used for impedance data fit.

The received EIS data from the electrode modification process was fitted to the Randles circuit (Scheme 2). The R_e_[CPE(R_ct_Z_W_)] equivalent electrical circuit expresses electrolyte-electrode interfacial and consists of electrolyte resistance (R_e_) combined parallel with the constant phase element (CPE) and an impedance Faradaic reaction (charge transfer resistance, R_ct_). Diffusion in the low frequencies’ region is presented as the Warburg element (Z_W_) [46].

The R_ct_ parameter is the most important for the impedance signal to analyze [47], thus this parameter was chosen as the evaluation of bacteria detection. The percentage of R_ct_ changes were calculated from the equation:

ΔR_ct_ = (R_ct 2_ − R_ct 1_)/R_ct 1_ × 100%, (1)

where R_ct 1_ is for bare Au, and R_ct 2_ is for the modification step. Calculated data is presented in Table 2. The data confirmed the modification success as the charge-transfer process was impeded, suggesting the increase of a biolayer thickness. The chemisorption of 4-ATP resulted in a 66% increase of R_ct_ compared to the bare electrode. The GA linkage resulted in a 132% increase of R_ct_ compared to the bare electrode. Subsequent increases derived from protein immobilization, giving a strong increase of 491% and 713% for anti-Psl and BSA, respectively.

### 3.3. Psl Bacteria Identification by qPCR

*Psl* identification was performed by qPCR amplification according to the method described in the Experimental section. The pathogen was detected in all samples, as shown in Table 3. Seven *Psl* strains and positive control (17 ng DNA) were checked as positive, three negative controls were confirmed as negative. By determining the standard curve (Figure 1), the number of viable bacteria in the tested samples was calculated. The study showed bacterial availability at the level of 0.47 to 1.17 × 10^9^ CFU/mL.

### 3.4. Psl Bacteria Detection on Developed Electrochemical Immunosensor

**Figure 3:**
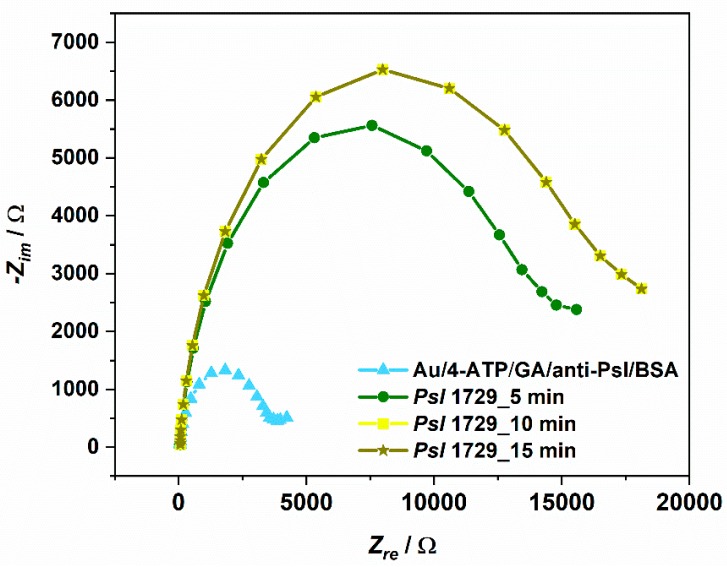
EIS measurements of biosensor response in time towards positive sample: *Psl* strain 1729. Registered in 5 mM K_3_[Fe(CN)6]/K_4_[Fe(CN)_6_]/0.1 M KCl.

The developed sensing system was applied for the detection of *Psl*. Firstly, the reference *Psl* strain 1729 was incubated on the Au/4-ATP/GA/anti-Psl/BSA electrode, and impedance spectra were recorded in time. The sensor response was checked in time cycles of 5, 10, and 15 min. Figure 3 presents the sensor response on *Psl* 1729. The strong change of EIS characteristic was observed after 5 min incubation. The baseline (BSA-modified electrode) drifted between electrodes due to nonrepetitive gold structures, thus the sensor response was presented in the relative impedance change. The percentage of R_ct_ changes were calculated from the equation:

ΔR_ct_ = (R_ct S_ − R_ct B_)/R_ct B_ × 100%, (2)

where R_ct S_ is for sample, and R_ct B_ is for Au/4-ATP/GA/anti-Psl/BSA.

The charge-transfer resistance value increased 281.8% over the Au/4-ATP/GA/anti-Psl/BSA measurement due to the presence of *Psl*. The second measurement after 10 min incubation resulted in the following R_ct_ increase of 334.52%. The third spectra after 15 min incubation practically remained unchanged compared to 10 min incubation. The results suggest that the binding of *Psl* cells to the antibodies creates a barrier for the electrochemical process. The redox probe had partly hindered access to the electrode surface resulting in a notable increase in the R_ct_ value. Moreover, similar spectra for 10 and 15 min incubation suggest that after 10 min the system was in equilibrium, which means that all antibodies active sites were saturated with antigen. The 10 min incubation was chosen as detection time, and the percentage change of the R_ct_ parameter was chosen as the sensor response towards samples.

**Figure 4:**
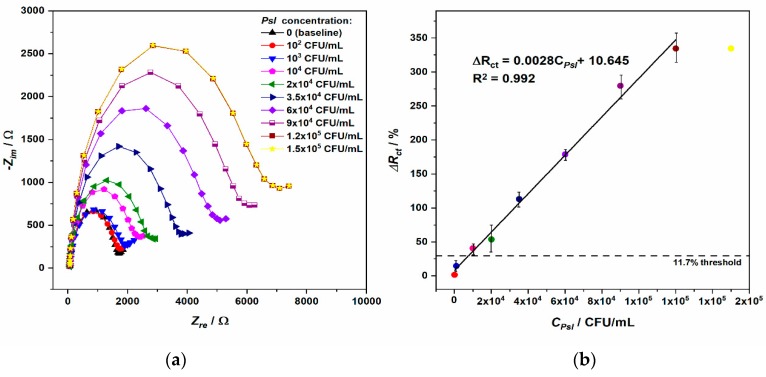
The linearity of immunosensor response on *Psl*: (**a**) Impedance spectra for Au/4-ATP/GA/anti-Psl/BSA electrode incubated with the series of *Psl* dilution. Registered in 5 mM K_3_[Fe(CN)_6_]/K_4_[Fe(CN)_6_]/0.1 M KCl; (**b**) the linear relation between Charge Transfer Resistance (R_ct_) change values and bacteria concentration. Error bars indicate the measurement standard deviations.

The next step of immunosensing system tests involved different concentrations of *Psl* for linear response and LOD determination. The series of eight bacterial concentrations expressed in CFU/mL were checked. Figure 4a displays the impedance changes obtained with Au/4-ATP/GA/anti-Psl/BSA electrode incubation with different concentrations of *Psl*. There is a progressive increase in sensor response with the increased *Psl* amount. The results indicate a significant impedance difference between *Psl* concentrations due to the increasing number of *Psl* cells which bind to immunosensor. At a concentration of 100 CFU/mL, the impedance change from a bacterial sample was on the negative control level. The smallest positive R_ct_ change was for 1 × 10^3^ CFU/mL (14.68%) and the highest for 1.2 × 10^5^ CFU/mL (334.52%). No more increase was observed with the subsequent incubations with a higher concentration of *Psl*. The linear relationship between the impedance change (R_ct_ change) and *Psl* concentration describes the equation:

ΔR_ct_ = 0.0028C*_Psl_* + 10.645, (3)

with an R^2^ coefficient of 0.992 and is presented in Figure 4b.

The sensitivity was established as the lowest quantity of *Psl* detectable in the assay (LOD). LOD was calculated from the impedance signal deriving from negative control (nontarget *Haemophilus influenzae* bacteria (*Hin*, results described below)). The *Hin* R_ct_ change signal was multiplied by three giving LOD of 11.7%, which equals 377 CFU/mL of *Psl*. Table 4 presents in detail the comparison of various detection methods of *Pseudomonas syringae* species mentioned in this article with the proposed electrochemical biosensor. According to the standard PCR method, which detects bacterial cells, this assay is less sensitive than real-time qPCR [27]. The real-time qPCR LOD is 1.3 times lower, but these values are very similar, in the same order of magnitude. However, the developed biosensor is more sensitive than PCR variation–loop-mediated isothermal amplification (LAMP) [28] and detects 29.7 times less *Pseudomonas syringae* cells.

**Figure 5:**
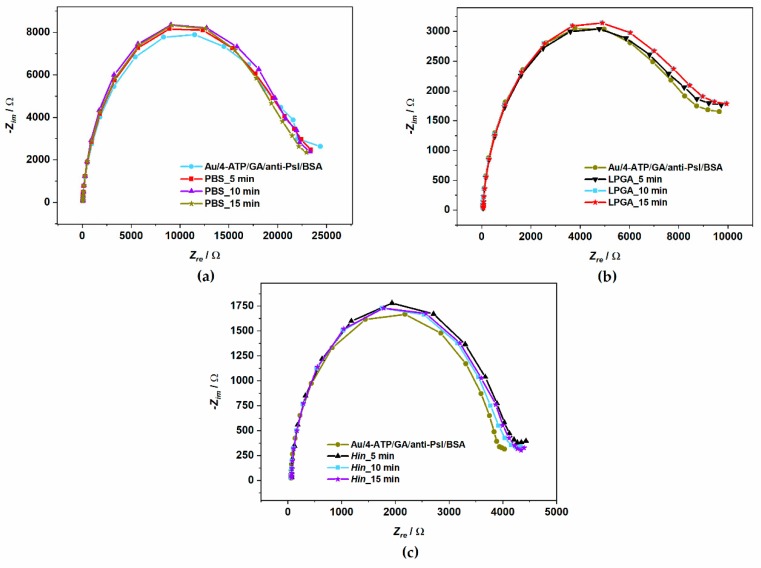
EIS measurements of biosensor response in time towards negative samples: (**a**) PBS, (**b**) LPGA medium, (**c**) *Hin* nontarget bacteria. Registered in 5 mM K_3_[Fe(CN)_6_]/K_4_[Fe(CN)_6_]/0.1 M KCl.

The last step of sensor evaluation was repeatability/reproducibility and selectivity studies. For the repeatability determination, one Au/4-ATP/GA/anti-Psl/BSA sensor was freshly prepared and used for the triplicate measurement of reference *Psl* strain 1729 (concentration of 10^4^ CFU/mL). For the reproducibility determination, three separately prepared Au/4-ATP/GA/anti-Psl/BSA sensors were used for the analysis of the same probe (*Psl* 1729, 10^4^ CFU/mL). The percentage changes of impedance in the presence of *Psl* 1729 on one sensor resulted in the average relative standard deviation (RSD) value of 1.74%. For three separate sensors, the average RSD resulted in 3.45%. For the selectivity studies, the series of negative controls were tested to exclude any unspecific responses. The negative PBS buffer used for bacteria dilution, LPGA bacterial medium, and nontarget *Hin* bacteria were chosen. All the samples were separately incubated on electrodes, and the series of measurements were recorded, similarly to the detection of positive *Psl* 1729. Figure 5 presents the sensor response towards all negative samples.

**Figure 6:**
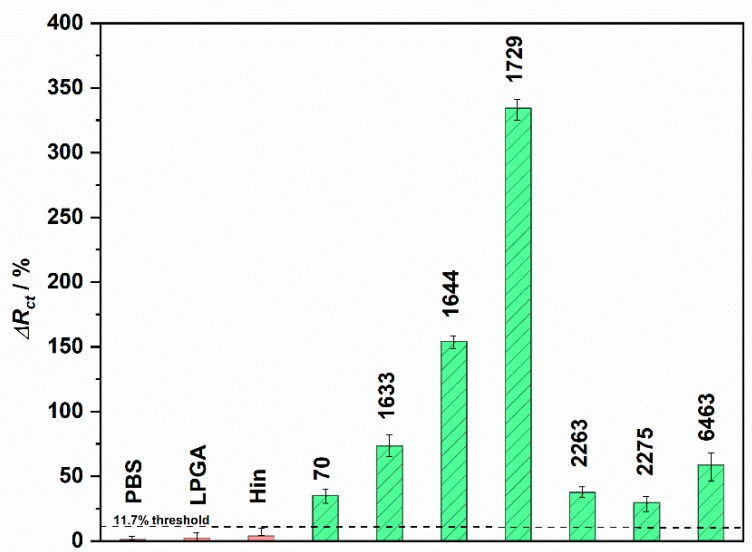
The specificity of the immunosensor for the detection of *Pseudomonas syringae* pv. *lachrymans*. Red (nonpatterned) bars are negative samples, green (patterned) bars are positive samples. Error bars indicate the measurement standard deviations.

From Figure 5, all the negative samples did not change the character of impedance spectra. Negligible R_ct_ increase was observed after 5 min incubation; thus, samples were further incubated in 5 min cycles until the stability of the system. In all cases, the repeatable EIS was after 10 min. similarly to positive *Psl* 1729 strain, confirming the chosen detection time. The R_ct_ increases were 1.3% and 2.3%, and for PBS and LPGA bacterial medium, respectively, compared to Au/4-ATP/GA/anti-Psl/BSA. The highest R_ct_ increase was 3.9% for nontarget *Hin* bacteria. For selectivity determination, except for negative control, seven PCR-confirmed strains of *Psl* were used as positive samples and examined on immunosensor. Detailed results of all tested samples were presented in the percentage R_ct_ change in Figure 6.

The 11.7% of R_ct_ change (LOD) was established as a threshold for the distinction between positive and negative samples in this assay. Three negative controls did not exceed this threshold, where all seven *Psl* strains confirmed by PCR were also tested as positive in the immunoassay giving visibly more significant impedance change. The strongest interaction with the anti-Psl antibodies had strain no. 1729 (334.52%), and the weakest strain no. 2275 (29.48%). According to obtained results, the sensor was highly specific for the detection of *Psl* bacteria and not giving false-positive results for PBS buffer, medium, and other bacteria.

## 4. Discussion

This work presented the design and characterization of anti-Psl antibodies immobilization onto gold electrodes for impedimetric detection of *Psl* bacteria. The development of the platform consisted of the 4-ATP self-assembled monolayer, glutaraldehyde crosslinker, antibodies, and BSA proteins. Bacterial detection relied on the impedance changes (increases) caused by *Psl* cells binding onto the Au/4-ATP/GA/anti-Psl/BSA surface. The assay could detect *Psl* at concentrations as low as 337 CFU/mL with a linear detection range 10^3^–1.2 × 10^5^ CFU/mL (R^2^ = 0.992). The sensitivity was in the same order of magnitude as the standard method (LOD for real-time qPCR was 1.3 times lower). Simultaneously, the assay was 30 times more sensitive than the LAMP method, which is an isothermal alternative for PCR. The sensor was proved to detect seven *Psl* strains. The specificity was evaluated using negative controls: PBS buffer, LPGA medium, and nontarget *H. influenzae* bacteria. There were no significant impedance changes when probes were incubated (all did not exceed the LOD of 11.7% R_ct_ change), which evidenced the sensing specificity towards *Psl*. The total detection time was 10 min.

The ease of assay preparation, label-free detection, and miniaturization possibilities qualifies it for the portable biosensing system. There is a continuous demand for simple analytical methods for the determination of many biochemical analytes. Assays based on antibody–antigen interactions are promising thanks to high specificities and sensitivities. The in-field monitoring of many crops for *Psl* infection risk is possible due to the accessibility of target analyte (whole bacteria cells). The chosen EIS detection method was fast, nondestructive for samples, and required low currents (battery mode). In the future, authors want to examine *Psl* directly from infected species, e.g., cucumber leaves, and implement the developed method on disposable electrodes.

## Figures and Tables

**Table 1:** Bacterial strains used in the experiment.

Scientific Name.	Collection No.	Geographic Origin
*Pseudomonas syringae* pv. *lachrymans*	70	France
*Pseudomonas syringae* pv. *lachrymans*	1633	Canada
*Pseudomonas syringae* pv. *lachrymans*	1644	Hungary
*Pseudomonas syringae* pv. *lachrymans*	1729	Denmark
*Pseudomonas syringae* pv. *lachrymans*	2263	Romania
*Pseudomonas syringae* pv. *lachrymans*	2275	Romania
*Pseudomonas syringae* pv. *lachrymans*	6463	Hungary

**Table 2:** Fitted data values for Randles circuit elements.

Modification Step	R_e_ [Ω]	R_ct_ [Ω]	CPE [µFΩ^−1^s^n^]	n *
Au	64.5	83.6	6.25	1.007
Au/4-ATP	54.3	138.4	6.61	0.850
Au/4-ATP/GA	55.0	194.0	3.14	0.889
Au/4-ATP/GA/anti-Psl	55.0	494.8	1.19	0.949
Au/4-ATP/GA/anti-Psl/BSA	62.1	679.4	1.08	0.954

* n, exponent of CPE parameter.

**Table 3:** Psl strains verification by qPCR.

Sample	Ct	CFU/mL	Test Result
Psl 70	14.07	1172325569	positive
Psl 1633	15.07	642100427.8	positive
Psl 1644	14.87	724255855.1	positive
Psl 1729	15.06	634377828.5	positive
Psl 2263	15.59	469488930.4	positive
Psl 2275	15.31	555708969.5	positive
Psl 6463	15.09	645964611.3	positive
17 ng DNA (positive control).	10.21	2629019000	positive
PBS	-	-	negative
LPGA medium	-	-	negative
*Haemophilus influenzae*	-	-	negative

**Table 4:** The comparison of *Pseudomonas syringae* detection methods.

Method	Target Analyte	LOD	Range of Determination	Year	Ref
Real-time qPCR	Bacterial DNA	260 CFU/mL	271–6.94 × 10^7^ CFU/mL	2019	[27]
LAMP	Bacterial cells	10^4^ CFU/mL	10^4^–10^8^ CFU/mL	2017	[28]
ELISA	Syringopeptins	0 ± 05 µg/well	0 ± 1–10 µg	1999	[29]
Colorimetric	Bacterial DNA	15 ng/μL	90–200 ng/L	2013	[37]
RPA + DPV	Bacterial DNA	21.4 pM	21.4 pM–2.140 µM	2017	[38]
**EIS.**	Bacterial cells	337 CFU/mL	1 × 10^3^–1.2 × 10^5^ CFU/mL	2019	This work

## Supplementary Materials

The following are available online at https://www.mdpi.com/1424-8220/19/24/5411/s1, Figure S1: Cyclic voltammetry spectra of gold electrode cleaning in 0.5 M H_2_SO_4_ in potential range from −0.1 to 1.5 V (30 scans) with a scan range of 100 mV/s.
