# Supplementary material for: Detection of the Plant Pathogen Pseudomonas Syringae pv. Lachrymans on Antibody-Modified Gold Electrodes by Electrochemical Impedance Spectroscopy
Source: Sensors (Basel). 2019 Dec 9;19(24):5411. doi: 10.3390/s19245411 (PMC6960962; doi:10.3390/s19245411)
Supplement: Supplementary file 1 [file sensors-19-05411-s001.pdf]

Supplementary Material

Detection of the Plant Pathogen *Pseudomonas Syringae* pv. *Lachrymans* on Antibody-Modified Gold Electrodes by Electrochemical Impedance Spectroscopy

Zofia Cebula [1], Sabina Żołędowska [1,2,*], Karolina Dziąbowska [2], Marta Skwarecka [1], Natalia Malinowska [2], Wioleta Białobrzeska [2], Elżbieta Czaczyk [2], Katarzyna Siuzdak [3], Mirosław Sawczak [3], Robert Bogdanowicz [4] and Dawid Nidzworski [1,2]

[1] Institute of Biotechnology and Molecular Medicine, 3 Trzy Lipy St., 80-172 Gdańsk, Poland; zofia.cebula@ibmm.org.pl (Z.C.); marta.spibida@etongroup.eu (M.S.); dawid@etongroup.eu (D.N.)
[2] SensDx, 14b Postępu St., 02-676 Warszawa, Poland; karolina.dziabowska@etongroup.eu (K.D.); natalia.malinowska@etongroup.eu (N.M.); wioleta.bialobrzeska@etongroup.eu (W.B.); ela@etongroup.eu (E.C.)
[3] Polish Academy of Sciences, The Szewalski Institute of Fluid-Flow Machinery, The Centre for Plasma and Laser Engineering, 14 Fiszera St., 80-231 Gdańsk, Poland; ksiuzdak@imp.gda.pl (K.S.); mireks@imp.gda.pl (M.S.)
[4] Faculty of Electronics, Telecommunications and Informatics, Department of Metrology and Optoelectronics, Gdańsk University of Technology, 11/12 G. Narutowicza St., 80-233 Gdańsk, Poland; rbogdan@eti.pg.edu.pl
* Correspondence: sabina.zoledowska@etongroup.eu

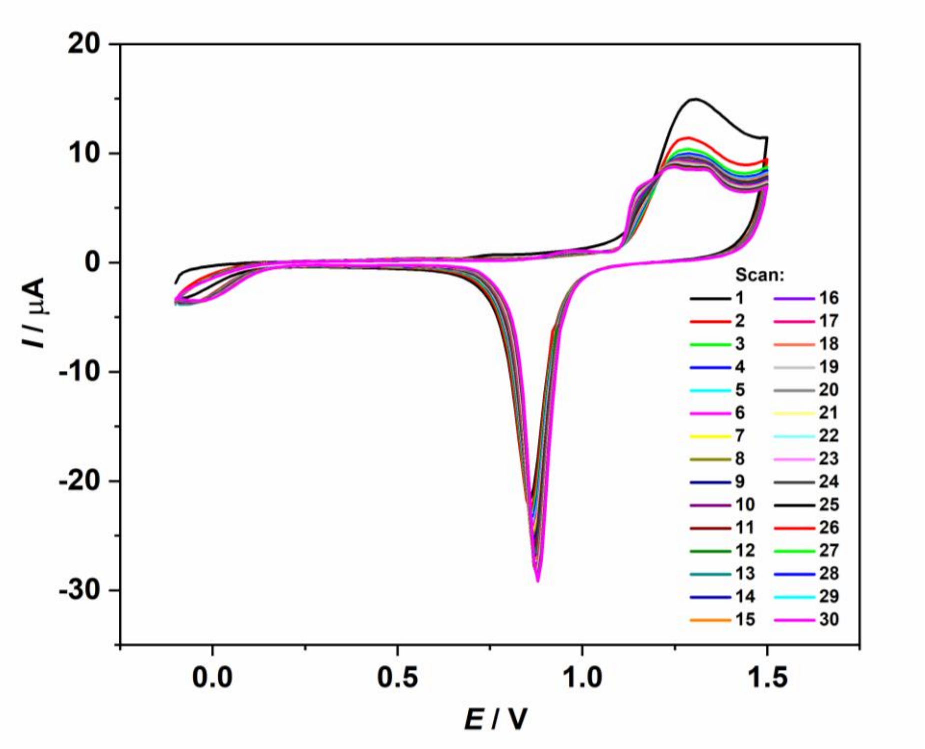

Figure S1. Cyclic voltammetry spectra of gold electrode cleaning in 0.5 M H_2SO_4 in potential range from -0.1 to 1.5 V (30 scans) with a scan range of 100 mV/s.
